# Toxic posterior segment syndrome with retinal vasculitis likely caused by intraocular cotton fiber after vitreoretinal surgery – a case report

**DOI:** 10.1186/s12886-023-03212-9

**Published:** 2023-11-16

**Authors:** Chaitra Jayadev, Aditi Gupta, Santosh Gopikrishna Gadde, Ramesh Venkatesh

**Affiliations:** Department of Retina and Vitreous, Narayana Nethralaya #121/C, 1st R block, Rajaji Nagar, 560022 Bangalore, India

**Keywords:** Toxic posterior segment syndrome, Ocular inflammation, Retinal vasculitis, Vitreoretinal Surgery, Steroids

## Abstract

**Background:**

Intraocular inflammation is common after anterior or posterior segment surgery. They typically manifest either as non-infectious inflammation of the anterior or posterior segment, known as toxic anterior or posterior segment syndrome (TPSS), or as sterile or infective endophthalmitis. In this report, we describe a rare case of TPSS following vitreoretinal surgery, presenting as hemorrhagic retinal vasculitis.

**Case presentation:**

A 58-year-old male diagnosed with a left eye acute rhegmatogenous retinal detachment underwent an uneventful primary pars plana vitrectomy with silicone oil endotamponade on the same day of presentation. At presentation, there were no signs of intraocular inflammation, and his visual acuity in the affected eye was 20/200.

**Results:**

The retina was well-attached with silicone oil in place on the first post-operative day. Along the inferior retinal periphery, a hemorrhagic occlusive vasculitis was observed. Clinical examination revealed retained intraocular cotton fiber along the inferotemporal quadrant over the retinal surface. In addition to the standard post-operative medications, a course of systemic steroids (40 mg per day of Prednisolone tablets) was started. At the end of the first post-operative week, clinical signs of hemorrhagic retinal vasculitis were beginning to resolve, and by the end of the fourth post-operative week, they had completely resolved.

**Conclusion:**

This report describes an unusual diagnosis of TPSS after vitreoretinal surgery, most likely due to the presence of an intraocular cotton fiber. This excessive inflammation of the posterior segment usually responds to a course of topical and systemic steroids.

## Background

Following an anterior or posterior segment surgery, intraocular inflammation is not uncommon [1]. Typically, they manifest as non-infectious inflammation of the anterior segment alone as toxic anterior segment syndrome, the posterior segment alone as toxic posterior segment syndrome (TPSS), or both, or as infective inflammation of the anterior or posterior segment as infective endophthalmitis [1]. Following vitreoretinal surgery, a number of reports describe anterior segment inflammation [2–4]. In general, TPSS denotes a distinct form of toxic intraocular syndrome characterized by non-infective inflammation confined solely to the posterior segment of the eye following surgical interventions involving the anterior or posterior segment of the eye [5]. TPSS was first reported by Patel et al. in 2020 as a result of intracameral locally compounded triamcinolone-moxifloxacin use following cataract surgery [6]. The manifestations of TPSS exhibit a greater degree of variability. The vitreous may present as either transparent or exhibit indications of vitritis. Additionally, the retina may display various characteristics such as hemorrhages, vasculitis, pigment epithelium lesions, macular edema, and, occasionally, optic disc pallor [5]. Every substance used in the surgical procedure could be a source of operative toxicity in TPSS. Among the many are those relating to the cleaning of micro cannulated instruments, intraocular infusions, enzymatic soaps, endotoxins, denatured viscoelastic, perfluorocarbons, silicone oil, and other endotamponade. Sahoo et al. reported a series of three cases of TPSS following vitreoretinal surgery for retinal detachment repair with the use of silicone oil as endotamponade. These cases presented with an occlusive retinal vasculitis-like picture immediately after surgery, with improvements in clinical findings and visual acuity following treatment with topical and systemic corticosteroids [7].

In this report, we describe a case of TPSS presenting with a picture resembling hemorrhagic occlusive retinal vasculitis, which was successfully treated with systemic and topical steroids, possibly due to an accidently introduced intraocular cotton fibre.

## Case presentation

**Fig. 1:**
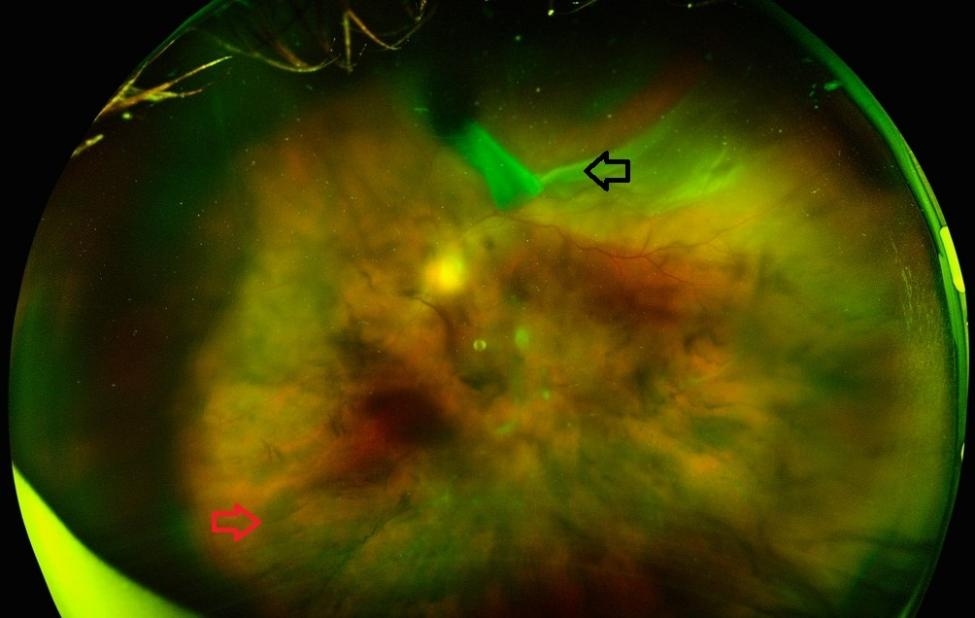
Pre operative fundus image of the left eye: Left eye fundus showing a superior large retinal tear (black arrow), retinal detachment and diffuse vitreous hemorrhage (red arrow)

A 58-year-old healthy man was referred to the retina department by the general ophthalmologist with a diagnosis of rhegmatogenous retinal detachment in the left eye. In the past four days, he was complaining of a sudden, painless decrease in vision in his left eye. His right eye visual acuity was light perception, while his left eye visual acuity was 20/200. His poor right eye vision was the result of a failed retinal reattachment procedure performed five years ago. His anterior segment examination and intraocular pressure examination were normal upon ocular examination. Both eyes exhibited pseudophakia with posterior intraocular lenses. The right eye fundus was vitrectomised and filled with silicone oil, with a thin, detached, atrophic retina and severe macular scarring. The fundus of his left eye revealed a total rhegmatogenous retinal detachment with a large superior retinal break extending posterior to the equator as well as an associated vitreous hemorrhage (Fig. 1). Through the visible retina, no clinical signs of retinal vasculitis or inflammation of the posterior segment were observed. On the same day, the patient underwent a 25-gauge primary pars plana vitrectomy with fluid-air exchange, endolaser to the retinal break, and silicone-oil endotamponade.

**Fig. 2:**
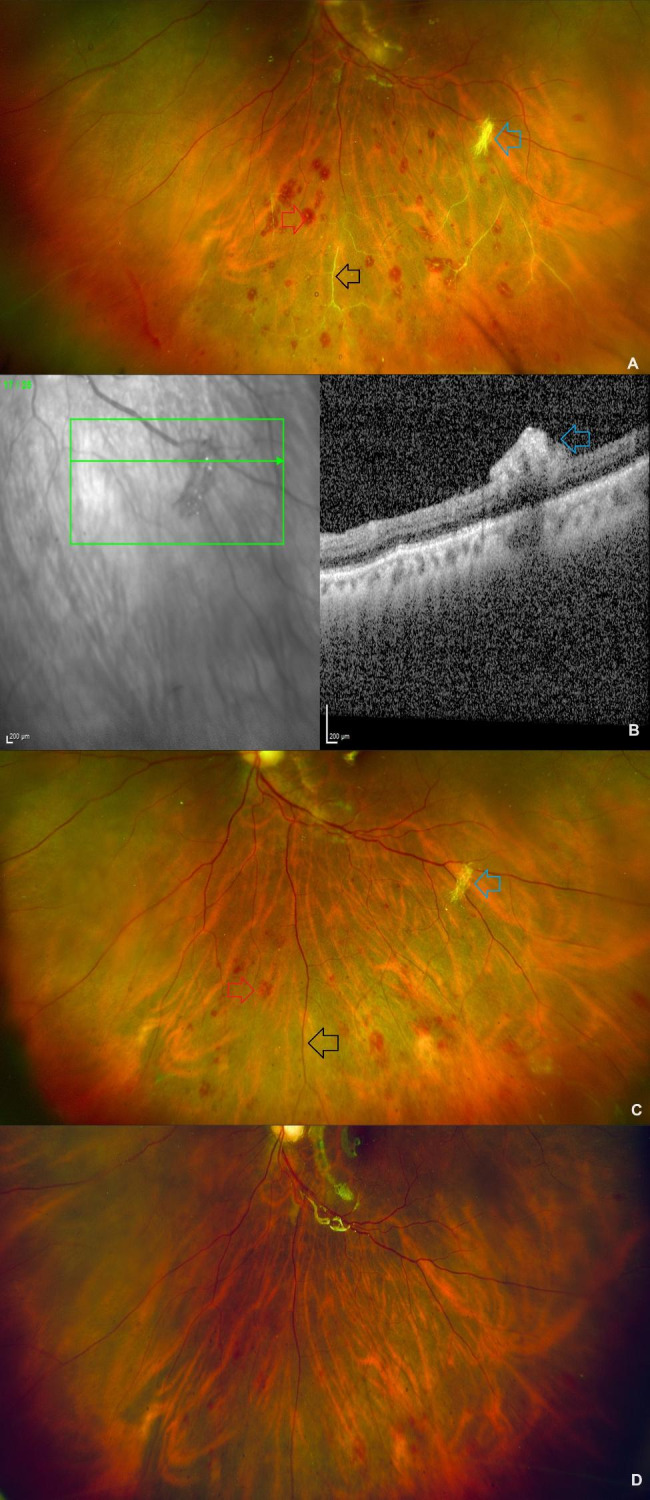
Sequential postoperative fundus images at day 1, week 1 and week 4 after vitreoretinal surgery: **A**: Postoperative day 1 fundus image of the left eye following vitrectomy and silicone oil tamponade showing vascular narrowing and sclerosed vessels (black arrow), pale centered hemorrhages (red arrow) and a ‘cotton fibres’ clump (blue arrow) along the inferotemporal arcade. **B**: Optical coherence tomography scan confirms the preretinal location of the cotton fibre. **C**: Postoperative week 1 fundus image of the left eye showing perfusion of the vessels (black arrow) and resolving hemorrhages (red arrow) with disintegration of the cotton fibres (blue arrow). **D**: Postoperative week 4 fundus image of the left eye on follow up showing complete resolution of the cotton fibres, with normalization of the blood vessels and resolution of majority of the retinal hemorrhages

The intraocular pressure was normal and there were no signs of anterior segment inflammation on the first postoperative day. The left eye’s fundus examination revealed a well-attached retina, a healthy optic disc, and silicone-oil in situ. There were sclerosed retinal vessels emerging from the equator and extending anteriorly along the inferior retinal periphery. Multiple blot-deep retinal hemorrhages were observed in the same region. A small number of these hemorrhages had white centres. In addition, a white lesion located preretinal along the inferotemporal arcade that resembled cotton fibres was observed upon clinical examination (Fig. 2A). An optical coherence tomography scan that passed through the lesion confirmed its preretinal location (Fig. 2B). The lesion exhibited minimal back shadowing. At this time, a few important differential diagnoses were considered. The most serious of these is the onset of acute infective endophthalmitis. A consultation with a uveitis specialist was arranged, and an aqueous chamber tap was performed immediately before beginning topical post-operative medications. The sample was sent for smear with Gram and KOH staining, as well as bacterial or fungal culture growth. The smear report revealed no microorganisms. Post-operative hypertension was measured at 130/80 mm hg, which could have been one of the causes of post-operative retinal vascular occlusion. The incident was reported to the hospital’s infection control committee. The committee looked into the remaining cases where the same batch of silicone oil or saline infusion was used. None of the other cases had similar clinical responses in the early post-operative period. Based on the observations of the uvea specialist and the hospital infection committee, an exaggerated post-operative, non-infective posterior segment inflammation was suspected. The removal of silicone oil, followed by the removal of cotton-fibre and silicone oil reinjection, was considered and discussed with the patient. The patient was not eager for an immediate second operation. Hence, in addition to regular post-operative topical medications, the patient received an additional course of oral systemic steroids (Tab Prednisolone 40 mg daily for 7 days). A week later, at the next follow-up appointment, retinal perfusion in the vessels was observed, and the retinal hemorrhages had begun to resolve with disintegration of the cotton fibres (Fig. 2C). The oral steroid dosage was tapered by 10 mg per week for an additional 3 weeks. The patient’s retinal vasculitis and retinal hemorrhages had reduced three weeks after the previous visit, along with a clear media, well-attached retina and silicone oil in place. The cotton fibre initially observed in the inferotemporal quadrant had disintegrated and disappeared (Fig. 2D). At this visit, the documented corrected distance visual acuity had improved to 20/40. At the most recent follow-up visit, eight weeks after the primary vitrectomy, the retinal findings had not worsened. Written informed consent was obtained from the patient for utilising his clinical details for this manuscript. Permission for using the patient data for this report was obtained from institutional review board and ethics committee (C/2023/06/03).

## Discussion and conclusion

To summarize, we present a case of exaggerated, non-infective posterior segment inflammation following retinal reattachment surgery that manifested as occlusive haemorrhagic retinal vasculitis on the first post-operative day. The presence of non-infective posterior segment inflammation, most likely caused by the toxicity induced by the retained intraocular cotton fibre, led to the diagnosis of TPSS.

The term TPSS refers to posterior segment inflammation of the eye caused by a reaction or toxicity to any substance used in the surgical procedure. Toxic posterior ocular reactions are usually caused by the use of intracameral adjuvants, such as those used in cataract surgery, or by the use of saline irrigating solution, silicone oil or gas tamponades, or perfluorocarbon liquid during vitreoretinal surgery [3, 4, 6, 7]. Similar hypersensitivity reactions have been noted following the use of intravitreal drugs such as anti-vascular endothelial growth factors or locally compounded antibiotics like vancomycin or even following the use of intraocular dyes such as indocyanine green or Brilliant blue G [8–12]. Substances related to the cleaning of micro cannulated instruments could also be responsible for TPSS. Toxic reactions in TPSS are acute hypersensitivity reactions that occur immediately after surgery and are potentially type 2 cytotoxic or type 3 immune-complex mediated reactions that respond effectively to systemic steroids [13].

Multiple factors, including the re-use of a previously cleaned vitrectomy set or instruments, saline infusion, intraocular air during fluid-air exchange, silicone oil endotamponade, and the presence of intraocular cotton fibre, may have contributed to the development of hypersensitivity in this case. The remaining cases, which were operated on the same day with the same vitrectomy set and instruments, as well as the same batch of silicone oil and saline infusion, showed no toxic intraocular reaction. As a result, we believe the hypersensitivity reaction was caused by an accidentally retained intraocular cotton fibre. In addition, the patient recovered from TPSS with a favourable visual outcome following the disappearance and disintegration of the intraocular cotton fibre and additional treatment with topical and systemic oral corticosteroids. This reinforces our belief that the toxic intraocular reaction was most likely caused by the retained intraocular fibre. However, a contaminated silicone oil vial or saline infusion solution cannot be completely ruled out as the likely source of TPSS in this case.

In conclusion, the appearance of a picture resembling occlusive retinal vasculitis following vitreoretinal surgery indicates exaggerated posterior segment ocular inflammation, which can be potentially vision-threatening in eyes with macular involvement. In such cases, identifying the trigger agent(s), particularly in TPSS clusters, is critical. Topical and systemic steroid treatment is usually effective.

## Data Availability

The datasets used and/or analysed during the current study are available from the corresponding author on reasonable request.

## Abbreviations

TPSS: Toxic posterior segment syndrome
